# Shifting the emphasis of brain health literacy from individuals to systems to reduce inequalities

**DOI:** 10.1002/alz.71624

**Published:** 2026-07-01

**Authors:** Timothy Daly

**Affiliations:** ^1^ Bioethics Program FLACSO Argentina Buenos Aires Provincia de Buenos Aires Argentina; ^2^ CNRS UMR 8011, Science Norms Democracy Sorbonne Université Paris Ile‐de‐France France

1

The recent study by Sandra Baez and colleagues^1^ showed that social disadvantage is more important than knowledge in dementia risk. Their work aligns with other related findings suggesting that social advantage confers protection against dementia onset in carriers of the apolipoprotein E4 (APOE4) risk gene for Alzheimer's disease.^2^ I argue that these findings should lead us to rethink brain health literacy by shifting the focus of literacy away from individuals and on to health systems.

Health literacy is usually defined as an ability of individuals, their “being able to access, understand, appraise, and use information and services in ways that promote and maintain good health and well‐being.”^3^ The brain health literacy of individuals and communities is certainly important, including in marginalized social groups.^4^ But given the growing scientific consensus demonstrating that factors affecting brain health beyond an individual's control are likely to be more significant than individual‐level factors,^5^ we should not focus exclusively on individuals’ responsibility to protect their own brains across the life course.^6^

Instead, we should embed our understanding of brain health inequalities into dementia research and policy^7^ as part of a whole‐population public health approach to dementia risk reduction.^8^ More literate health systems would promote public health policy that actively attempts to reduce brain health inequalities by focusing on deeper determinants of dementia risk rather than policy focused only on individuals and their behaviors that may inadvertently widen pre‐existing brain health inequalities.

In a nutshell, in light of the important work by Baez et al., the emphasis of brain health literacy should not only be on making individuals more knowledgeable about dementia risk factors, but on designing more literate health systems that promote action to reduce social disadvantage and health inequalities.

## CONFLICT OF INTEREST STATEMENT

The author declares no conflicts of interest. Author disclosures are available in the Supporting Information.

## FUNDING INFORMATION

The author has nothing to report.

## Supporting information



Supporting Information
